# Dual PI-3 kinase/mTOR inhibition impairs autophagy flux and induces cell death independent of apoptosis and necroptosis

**DOI:** 10.18632/oncotarget.6986

**Published:** 2016-01-22

**Authors:** Robert W. Button, Joseph H. Vincent, Conor J. Strang, Shouqing Luo

**Affiliations:** ^1^ Peninsula Schools of Medicine and Dentistry, Institute of Translational and Stratified Medicine, University of Plymouth, Research Way, Plymouth, UK

**Keywords:** autophagy, mTOR, PI3KC3, apoptosis, necroptosis

## Abstract

The PI-3 kinase (PI-3K)/mTOR pathway is critical for cell growth and proliferation. Strategies of antagonising this signaling have proven to be detrimental to cell survival. This observation, coupled with the fact many tumours show enhanced growth signaling, has caused dual inhibitors of PI-3K and mTOR to be implicated in cancer treatment, and have thus been studied across various tumour models. Since PI-3K (class-I)/mTOR pathway negatively regulates autophagy, dual inhibitors of PI-3K/mTOR are currently believed to be autophagy activators. However, our present data show that the dual PI-3K/mTOR inhibition (DKI) potently suppresses autophagic flux. We further confirm that inhibition of Vps34/PI3KC3, the class-III PI-3K, causes the blockade to autophagosome-lysosome fusion. Our data suggest that DKI induces cell death independently of apoptosis and necroptosis, whereas autophagy perturbation by DKI may contribute to cell death. Given that autophagy is critical in cellular homeostasis, our study not only clarifies the role of a dual PI-3K/mTOR inhibitor in autophagy, but also suggests that its autophagy inhibition needs to be considered if such an agent is used in cancer chemotherapy.

## INTRODUCTION

Macroautophagy (autophagy hereafter), is a bulk degradation system that mediates clearance of unwanted cytoplasmic constituents, including aberrant long-lived cytoplasmic proteins, intracellular pathogens and damaged organelles such as mitochondria [1-3]. Autophagy involves the formation of double-membrane structures called autophagosomes which engulf portions of cytoplasm. They are then trafficked to lysosomes where their contents are degraded after fusion [1]. During autophagosome-lysosome fusion, autophagosomes may directly fuse with lysosomes, or initially fuse with late endosomes to form amphisomes, which then subsequently fuse with lysosomes [4].

The ULK1/2-Atg13-FIP200 complex senses the signals for autophagosome initiation and stimulates the downstream class-III PI-3 kinase (PI-3K), Vps34 [5, 6]. Beclin 1-Vps34 complex generates PtdIns3P (PI(3)P), which is required for autophagosome/phagophore nucleation [7]. Autophagosome elongation involves two essential ubiquitin-like (UBL) conjugations: the conjugation of the UBL protein Atg12 to Atg5, and the conjugation of the UBL protein LC3 to phosphatidylethanolamine (PE). Atg5-Atg12 conjugation is catalysed sequentially by E1-like enzyme Atg7 and E2-like enzyme Atg10 [8]. The Atg5-12 conjugate (Atg5-12) is an E3 ligase to catalyse LC3-PE (LC3-II) conjugation that also requires E1-like Atg7 and E2-like Atg3 [9-11]. LC3-II, the lipidated form of LC3, is required for the expansion and completion of pre-autophagosomal membranes [12-14].

The ligation of growth factors to their receptors activates the class-I PI-3K and AKT kinase, that in turn activate mammalian target of rapamycin (mTOR) by inhibition of mTOR negative regulator TSC2, thus stimulating cell growth and proliferation [15]. mTOR has a number of downstream biological effects, including protein translation via phosphorylation of 4E-BP1 and p70 S6 kinase [16]. Under nutrient replete conditions, the TOR kinase negatively regulates autophagosome formation [17] via phosphorylation of Atg13 and ULK1, the upstream signals of autophagy initiation [18]. Autophagy is activated by starvation via multiple signals, including mTOR inhibition, and AMP-activated protein kinase activation [19].

The PI-3K/mTOR pathway is over-activated in many cancer cells [20, 21]. Therefore, it is crucial to target this pathway to suppress growth and induce cell death in these cells for therapeutic purpose. Chemical compounds targeting both PI-3K and mTOR, such as PI-103 and NVP-BEZ-235, have been designed to better target the survival signaling pathway [22, 23]. These compounds have shown potent effects in inhibiting cell survival and promoting cell death [24, 25], as well as enhancing the efficacy of chemotherapy and radiotherapy when used in combination treatments [26, 27]. Currently, PI-103 has been tested for treating many cancer models, such as gliomas [25, 28], AML leukemia [29] and prostate and breast carcinomas [30], amongst others. NVP-BEZ-235 has been investigated in a number of clinical trials as a potential chemotherapeutic for solid tumours seen in breast cancer (ClinicalTrials.gov ID: NCT01495247) and renal cell carcinoma patients (NCT01453595), as well as castration-resistant forms of prostate cancer (NCT01717898) and pancreatic neuroendocrine tumours (NCT01628913). Such dual kinase inhibitors will be further exploited for cancer therapy, with new agents entering clinical trials [31].

Dual inhibitors of PI-3K/mTOR are currently viewed as autophagy activators [28, 32, 33] since they simultaneously inhibit class-I PI-3K and mTOR [25], both of which suppress autophagy. However, this notion may be an oversimplification, as different classes of PI-3Ks have opposing effects on autophagy [34]. Whilst the class-I PI-3K does indeed inhibit autophagy initiation [35], the class-III PI-3K (Vps34 in mammals) generates PI(3)P for autophagosome nucleation and promotes autophagy [4]. We unexpectedly found that rather than inducing autophagy, dual PI-3K/mTOR inhibition (DKI) strongly inhibits the later stage of autophagy. This effect appears to be the result of inhibition of the class-III PI-3K Vps34, which causes a defect to autophagosome maturation. Our present data suggest that autophagy inhibition by the dual inhibitors may contribute to their toxicity, thereby offering a mechanism of cell death induction by dual inhibitors of PI-3K/mTOR.

## RESULTS

### Dual inhibition of PI-3K/mTOR causes an increase in autophagosome numbers

**Figure 1:**
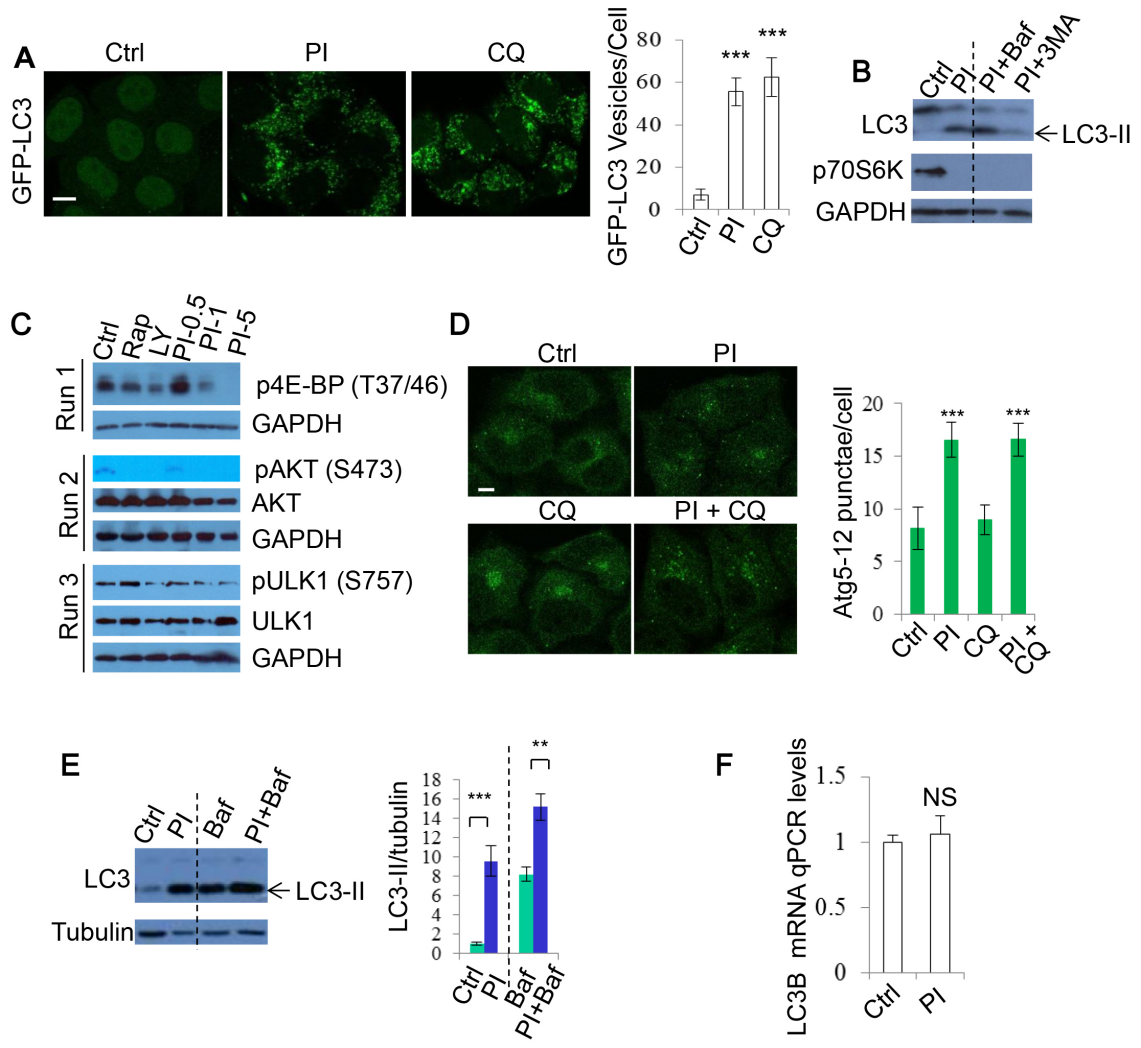
PI-103 causes autophagosome accumulation **A.** GFP-LC3 was transfected into HeLa cells. After 16 hours, cells were treated with vehicle (DMSO), PI-103 (PI) (5uM) or CQ (25uM). The cells were analysed after a further 20 hours. The numbers of GFP-LC3 vesicles representing autophagosomes are assessed (*n* = 30 cells per condition). Data are shown as mean ±sd. ***: *P* < 0.001. Scale bar: 20um. **B.** HeLa cells were treated with vehicle, PI (1uM), PI (1uM)+Baf (100nM), or PI (1uM)+ 3-MA (10mM) for 20 hours. Blots were probed with the indicated antibodies. **C.** HeLa cells were treated with vehicle, rapamycin (Rap) 2uM, LY294002 (LY) (10uM), and PI as shown for 20 hours, then analysed by immunoblot with the indicated antibodies. **D.** HeLa cells were treated with vehicle, PI (1uM) or/and CQ (25uM) for 20 hours, then fixed and stained with Atg12 antibody. Images were taken by confocal microscopy. The number of Atg12 puncta per cell were counted (*n* = 30 cells per condition). Data are presented as mean ±sd. Scale bar: 10um. **E.** HeLa cells were treated with vehicle, PI (5uM) for 20 hours with or without Baf (400nM) for 4 hours. Blots were probed with the indicated antibodies. Quantification is shown as mean LC3-II/tubulin. **: *P* < 0.01. **F.** HeLa cells were treated with vehicle or PI (5uM) for 20 hours. Isolated RNA was then analysed by qRT-PCR to detect LC3-B mRNA expression (experiments were performed in triplicate, with *n* = 3 per experiment). Data are shown as mean ±sd of relative mRNA levels (normalised to actin). NS: not significant.

As PI-3K/Akt/mTOR negatively regulates autophagy, DKI has been suggested to stimulate autophagy [28, 32, 33]. We first tested this by quantifying the numbers of GFP-LC3 positive vesicles exploiting DKI drug PI-103 treatment. The number of GFP-LC3 vesicles was markedly increased after PI-103 treatment in GFP-LC3 stably expressing HeLa cells, as reported previously [28], to an extent similar to the lysosomal degradation inhibitor Chloroquine (CQ) (Figure 1A). We observed the same effect in RT4-D6P2T neuronal Schwann (RT4) cells (Supplementary Figure 1). Autophagosome-associated protein LC3-II has been used as an autophagosome marker since the levels of LC3-II correlate with autophagosome numbers [1]. Likewise, PI-103 robustly increased LC3-II levels in these cells as detected by immunoblot. Of note, the effect was only slightly enhanced by the lysosomal inhibitor Bafilomycin A1 (Baf), but markedly reduced by the PI-3K inhibitor 3-methyladenine (3-MA), and phosphorylation of p70S6 kinase (p70S6K) (threonine 389), a prominent mTOR substrate, was inhibited by PI-103, confirming that PI-103 strongly inhibited mTOR kinase activity in our system (Figure 1B). We further assessed the effects of the drug treatment on a number of mTOR/PI-3K substrates (Figure 1C). Similarly, the phosphorylation of AKT (Serine 473) by PI-3 kinase and mTOR [36, 37], and that of ULK1 (Serine 757) by mTOR [18], were also decreased by PI-103 (Figure 1C). Of note, rapamycin treatment does not appear to significantly block the phosphorylation of ULK1 in our conditions. It was revealed that rapamycin inhibits S757 phosphorylation of ULK1 in short-term treatment (1 hour) condition [18]. This suggests that longer-term rapamycin treatment (20 hours) in our conditions may exert additional or feedback effect, leading to reduction in S757 phosphorylation of ULK1. Together, these data suggest that PI-103 induces the initiation of autophagosome formation, given that the phosphorylation of AKT and ULK1 blocks autophagosome synthesis [18, 36]. Indeed, PI-103 treatment led to an elevation in Atg5-12 puncta, as detected by Atg12 staining (Figure 1D).

Since autophagy encompasses the delivery of LC3-II-associated autophagosomes to lysosomes and their subsequent breakdown (‘autophagy flux’) [1, 38], increases in LC3-II can also be indicative of a blockade to autophagosome degradation. To test if PI-103 altered autophagy flux, we utilized Baf, that blocks lysosomal acidification and prevents subsequent autophagosome clearance [39]. Figure 1E shows that PI-103 massively increased the level of LC3-II (LC3B-II) (~10 fold) in HeLa cells. However, this increase (~2 fold) was largely weakened in the presence of Baf, suggesting that PI-103 may also inhibit lysosomal function or autolysosome formation. In accordance with this, qPCR analysis revealed that PI-103 treatment caused no significant alterations to LC3-B mRNA levels (Figure 1F). This indicates the drug-induced LC3-II increases are likely at the protein level, so potentially the result of impaired degradation.

### PI-103 blocks autophagic flux

**Figure 2:**
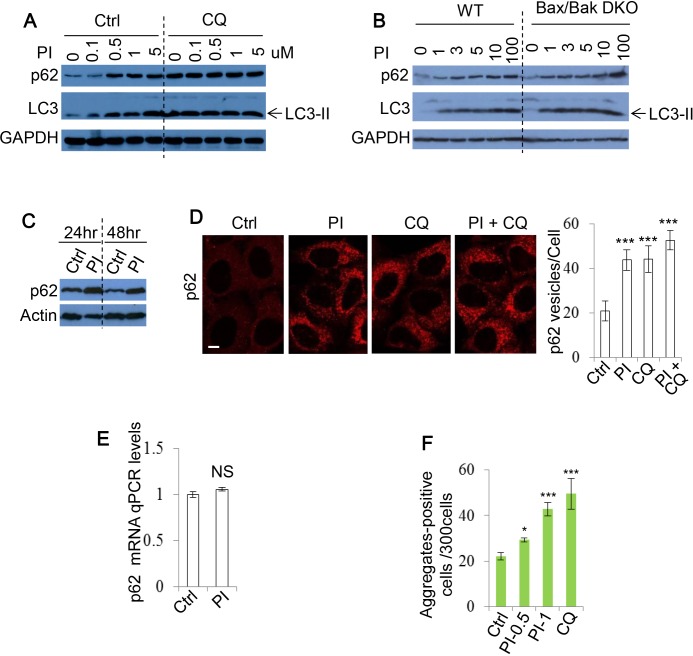
PI-103 blocks autophagic flux **A.** HeLa cells were treated with PI-103 (PI) as indicated with or without CQ (25uM) for 20 hours. Blots were probed with the indicated antibodies. **B.** WT and Bax/Bak DKO MEFs were treated with PI as indicated for 20 hours and subjected to immunoblot with the antibodies shown. **C.** HeLa cells were treated with vehicle and PI (1uM) at the indicated times and analysed by immunoblotting. **D.** HeLa cells were treated with vehicle, PI (1uM), CQ (25uM) or PI (1uM)+CQ (25uM) as indicated for 20 hours. Cells were fixed and stained for p62, and images taken by confocal microscopy. The numbers of p62 puncta per cell were counted (*n* = 30 cells per condition). Data are shown as mean ±sd. ***: *P* < 0.001. Scale bar: 10um. **E.** HeLa cells were treated with vehicle or PI (5uM) for 20 hours. RNA was isolated and analysed by qRT-PCR to detect p62 expression (experiments were performed in triplicate, with *n* = 3 per experiment). Data are shown as mean ±sd of relative mRNA levels (normalised to actin). NS: not significant. **F.** HeLa cells were transfected with GFP-Huntingtin (Htt)-72Q and treated with vehicle, PI (0.5 and 1uM) or CQ (25uM) 24 hours post transfection. Cells were fixed after a further 4 days, and the number of Htt aggregates-positive cells per 300 cells was recorded. Data are presented as mean ±sd. *: *P* < 0.05.

Our data unexpectedly suggested that DKI may impair autophagy. To explore this possibility further, we used additional methods of assessing autophagy flux. p62 recruits cargo to be engulfed by autophagosomes and is subsequently degraded by lysosomal enzymes after autophagosome-lysosome fusion [40]. As p62 is an autophagy substrate, increased autophagy levels are associated with p62 clearance. Consistently, we found that after PI-103 treatment, the clearance of p62 was impaired in HeLa cells (Figure 2A) and either wild-type (WT) or Bax/Bak double knockout (DKO) mouse embryonic fibroblasts (MEFs) [41, 42] (Figure 2B). The PI-103-induced decrease in p62 clearance was maintained at both 24 and 48 hours post drug treatment (Figure 2C). Similarly, the numbers of cytoplasmic p62 puncta observable by immunocytochemistry were elevated by PI-103, but not significantly enhanced when used in combination with CQ (Figure 2D). Additionally, no significant alterations to p62 mRNA levels were detectable during PI-103 treatment, indicating these increases occur at the protein level (Figure 2E). Taken together, these findings suggest that autophagy flux is inhibited by PI-103. We aimed to verify this using an alternative autophagy substrate. The Huntington's Disease protein, mutant huntingtin with expanded polyQ (mHtt), is known to form protein aggregates that are subject to autophagic clearance, and therefore can be used as another indicator of autophagy flux [43, 44]. We observed an increase in the number of mHtt aggregates after PI-103 addition, to an extent comparable to CQ (Figure 2F), providing further support for a role of DKI in inhibition of autophagy.

### PI-103 blocks autophagosome-lysosome fusion

**Figure 3:**
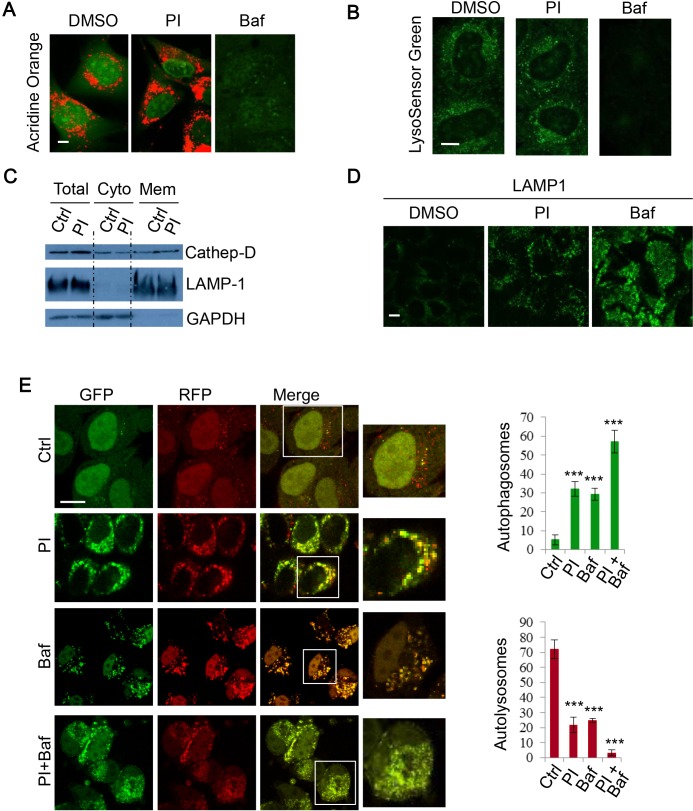
PI-103 blocks autophagosome-lysosome fusion **A.** RT4 cells were treated with vehicle, PI (1uM) or Baf (100nM) for 16 hours and then stained with acridine orange (AO) for 15 mins. Representative live cell images were taken on a confocal microscope. Scale bar: 5um. **B.** HeLa cells were treated with vehicle and PI (1uM) for 16 hours, or Baf (400nM) for 4 hours, and then stained with 1uM LysoSensor Green DND-189 for 30 minutes. Representative live cell images were taken on a confocal microscope. Scale bar: 10um. **C.** HeLa cells were treated with vehicle or PI (1uM) for 24 hours and fractionated to separate cytosolic and membrane components. Lysates were then subjected to SDS-PAGE and probed with the indicated antibodies. **D.** HeLa cells were treated with vehicle, PI-103 (PI) (1uM) or Baf (100nM) for 24 hours, then fixed and stained for LAMP1. Representative images were taken on a confocal microscope. Scale bar: 10um. **E.** mRFP-GFP-LC3 stably expressing HeLa cells were treated with vehicle, PI (1uM), Baf (100nM) or PI (1uM)+Baf (100nM) for 20 hours. Cells were then fixed and confocal images were acquired. The numbers of autophagosomes (green vesicles) and autolysosomes (red vesicles minus green vesicles) were assessed with automated Cellomics microscopy (*n* = 12). Data are shown as mean ±sd. ***: *P* < 0.001. Scale bar: 20um.

As the DKI drug PI-103 appeared to disrupt autophagy flux, we next sought to investigate whether this effect was the result of changes to lysosome acidity or integrity. Acridine orange (AO) is a lysosomotropic metachromatic fluorochrome that emits red fluorescence under acidic pH, which is normally in late endosomes/lysosomes, and green fluorescence otherwise [45]. Thus, reduced red fluorescence may reflect the reduced integrity of lysosome membranes. To further characterise the effect of PI-103 on lysosomal mass or membrane integrity, AO was used to stain lysosomes in RT4 cells treated with PI-103. In Figure 3A, we consistently observed that PI-103 induced lysosomal accumulation, but did not compromise lysosomal membrane integrity, whereas Baf treatment, which results in a defect in lysosomal acidification [39], was associated with a markedly reduced signal (Figure 3A). Similar observations were made with an alternative pH sensitive dye, LysoSensor Green. Whilst Baf caused a notable reduction in acidic vesicles, lysosomes were retained with PI-103 treatment (Figure 3B). Cell fractionation experiments confirmed that PI-103 did not compromise the integrity of lysosome membranes, since the pool of lysosomal cathepsin D was not reduced in PI-103-treated cells, compared to the control cells (Figure 3C). Although the acidity and integrity of lysosomes appeared to remain intact during PI-103 treatment, we noted a degree of vesicle accumulation with AO (Figure 3A). We confirmed the lysosome accumulation effect using the lysosomal membrane protein LAMP1, with a stronger signal seen in PI-103-treated cells compared to that in control cells (Figure 3D). Following this, we explored if autophagosome-lysosome fusion was impaired. To do this, we employed mRFP-GFP-LC3 stably expressing cells to monitor autophagosome synthesis and autophagosome-lysosome fusion, as autophagosomes appear yellow (with green and red) and autolysosomes as red vesicles, since the low lysosomal pH quenches GFP more quickly than mRFP [46]. Following PI-103 treatment, the number of autophagosomes increased, whilst autolysosomes markedly decreased (Figure 3E), indicating a blockade to autophagosome-lysosome fusion/autophagosome maturation. The decrease in autophagosome-lysosome fusion was comparable to that following Baf treatment (Figure 3E). Therefore, these findings suggest PI-103 perturbs autophagy flux.

### NVP-BEZ-235 also inhibits autophagy flux

**Figure 4:**
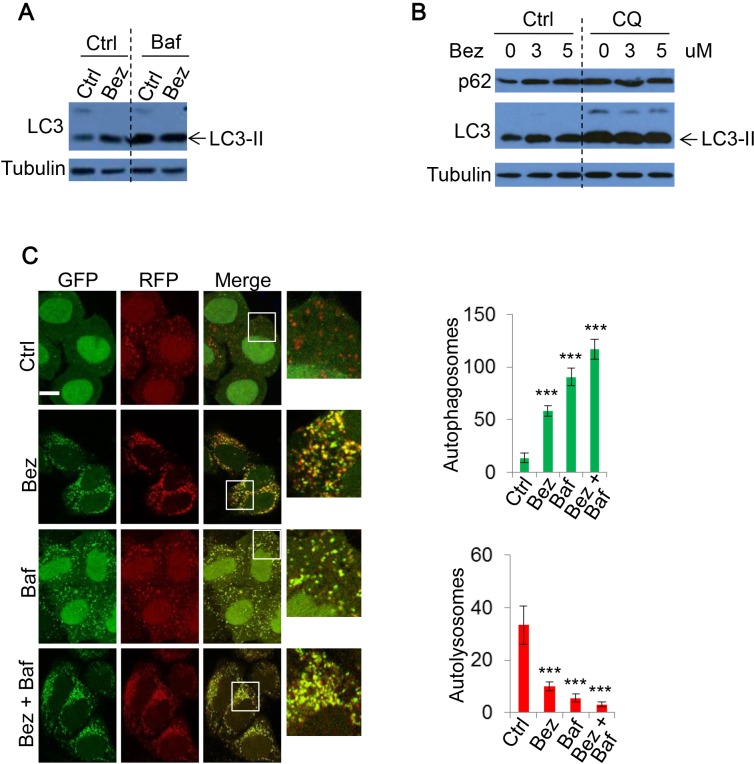
The effect of NVP-BEZ-235 on autophagy **A.** HeLa were treated with vehicle, NVP-BEZ-235 (BEZ-235) (0.5uM) for 20 hours with or without Baf (400nM) for 4 hours. Cell lysates were subjected to immunoblotting with the indicated antibodies. **B.** HeLa cells were treated with BEZ-235 at indicated concentrations, with or without CQ (25uM) for 20 hours. The lysates were analysed by immunoblot. **C.** mRFP-GFP-LC3 stably expressing HeLa cells were treated with vehicle or BEZ-235 (0.5uM) for 20 hours, and Baf (400nM) for 4 hours. Cells were then fixed and confocal images were acquired. The numbers of autophagosomes (green vesicles) and autolysosomes (red vesicles minus green vesicles) were assessed (*n* = 30 cells per condition). Data are shown as mean ±sd. ***: *P* < 0.001. Scale bar: 10um.

Our data indicated that PI-103 inhibits autophagy flux. To assess the consistency of this DKI-induced defect on autophagic flux, we further examined another prominent PI-3K/mTOR dual inhibitor, NVP-BEZ-235 (BEZ-235). Treatment with BEZ-235 increased LC3-II levels (Figure 4A). Similarly, BEZ-235 did not further increase LC3-II levels in the presence of Baf (Figure 4A), suggesting that BEZ-235 blocks autophagosome-lysosome fusion, rather than solely increase autophagosome biogenesis. BEZ-235 also prevented autophagic substrate p62 degradation (Figure 4B), lending further support to BEZ-235 inhibiting autophagic flux.

To further test whether DKI impairs autophagosome-lysosome fusion and subsequently causes autophagosome accumulation, we again utilized mRFP-GFP-LC3 stably expressing cells for BEZ-235 treatment. As with PI-103, BEZ-235 treatment caused autophagosome number to largely increase, while autolysosome number dramatically decreased, indicating autophagosome-lysosome fusion was blocked (Figure 4C). The changes were comparable to those resulting from the lysosome inhibitor Baf (Figure 4C). Therefore, DKI actually blocks rather than activates autophagic flux, although autophagosome accumulation was observed.

### Vps34 is required for autophagosome-lysosome fusion

**Figure 5:**
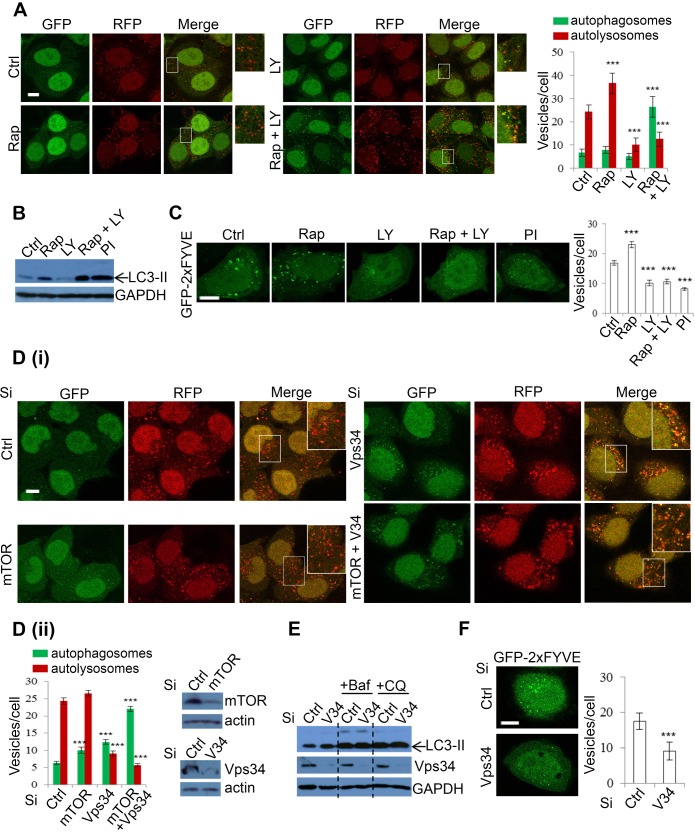
Inhibition of Class III PI-3K decreases autophagosome-lysosome fusion **A.** mRFP-GFP-LC3 stably expressing HeLa cells were treated with vehicle, rapamycin (Rap) (2uM), LY294002 (LY) (10uM), Rap (2uM)+LY (10uM) for 24 hours and the numbers of autophagosomes and autolysosomes were counted. Data are shown as mean ±sd (*n* = 30 cells per condition). ***: *P* < 0.001. Scale bar = 10um. **B.** HeLa cells were treated with Rap and LY as in A and PI-103 (PI) (1uM) for 24 hours and analysed by immunoblot with the indicated antibodies. **C.** HeLa cells were transfected with GFP-2xFYVE. 24 hours after transfection, cells were treated as in B and then fixed after a further 24 hours. Images were taken by confocal microscopy. The number of GFP-2xFYVE puncta per cell were counted (*n* = 30 cells per condition). Data are shown as mean ±sem. Scale bar: 10um. **D.** mRFP-GFP-LC3 stably expressing HeLa cells were transfected with control, mTOR and Vps34 siRNA as indicated for 48 hours (i). Autophagosomes (green vesicles) and autolysosomes (red vesicles minus green vesicles) were counted (*n* = 30 cells per condition) (ii­). All counts are shown as mean ±sd. Scale bar = 10um. Knockdown efficiency was confirmed with immunoblot. **E.** HeLa cells were transfected with control or Vps34 siRNA. 24 hours post transfection, cells were treated with vehicle, Baf (100nM) and CQ (25uM) for a further 20 hours, then analysed by immunoblot with the indicated antibodies. **F.** Control or Vps34 siRNA was co-transfected with GFP-2xFYVE for 48 hours. Cells were fixed and FYVE vesicles were quantified (*n* = 30). Data are shown as mean ±sd. Scale bar: 10um.

As the lysosomal effects of DKI had not been previously reported, we were interested in determining the mechanism by which these effects were mediated. To dissect DKI-targeted autophagy pathways, we employed a pharmacological strategy by using the single treatments of mTOR inhibitor rapamycin (Rap), and PI-3K inhibitor LY294002 (LY), or the combinatory treatment of Rap+LY. Both Rap and LY are widely used in autophagy research, with Rap inducing autophagosome synthesis [47], and LY inhibiting this process [48]. We first applied the treatment strategy to mRFP-GFP-LC3 cells. Whilst Rap incubation caused a significant increase in autolysosomes, indicating an increased autophagy flux, LY treatment caused a decline in these vesicles. The reduction of autolysosomes was similar between LY and Rap+LY combination treatments (Figure 5A). However, the Rap+LY combination treatment enhanced autophagosome numbers (Figure 5A). These data suggested that whilst loss of mTOR with DKI leads to elevated autophagosome synthesis, PI-3K ablation may result in the blockade to autophagosome maturation and subsequent degradation. Consistently, Rap caused an increase in the levels of LC3-II whereas LY led to a decrease (Figure 5B). However, the combination of both agents resulted in a further increase in LC3-II levels compared to Rap alone, to a magnitude similar to PI-103 (Figure 5B). This suggests that PI-3K inhibition is critical in the suppression of autophagy flux. We postulated that the effect of PI-3K inhibition on autophagosome maturation is attributable to loss of PI(3)P, since PI(3)P is a product of the Class III PI-3K [49] and has been associated with roles in autophagosome synthesis [7], as well as vesicle trafficking, endosome maturation and autophagosome maturation [50-53]. GFP-2xFYVE has been used as a bona fide probe to detect intracellular PI(3)P levels [54], thus we assessed the effects of drugs on PI(3)P levels in cells expressing GFP-2xFYVE. As expected, FYVE puncta were increased with rapamycin. However, PI-3K inhibition, with either LY or PI-103, caused a reduction in both number and size of FYVE dots, with more diffuse FYVE signal (Figure 5C). With PI-3K pharmacological inhibition and the subsequent reduction in PI(3)P seemingly disrupting autophagy flux, we aimed to fortify this observation with a more specific genetic approach by knocking down mTOR, Vps34 (kinase subunit of class III-PI-3K complex) or both mTOR and Vps34, and assessing autophagosome maturation in mRFP-GFP-LC3 cells. As expected, mTOR knockdown caused increases in the numbers of autophagosomes and autolysosomes (Figure 5D), which are plausibly the result of increased autophagosome synthesis and maturation caused by decreased mTOR activity. Interestingly, Vps34 knockdown increased LC3-positive vesicles (Figure 5D), unlike LY treatment (Figure 5A), presumably because LY does not solely inhibit Vps34 activity, but targets broader PI-3Ks. Vps34 knockdown appeared to block autophagosome maturation as autophagosome-lysosome fusion was compromised in Vps34 knockdown cells (Figure 5D). Both mTOR and Vps34 knockdown caused a further increase in autophagosome numbers (Figure 5D). Immunoblot analysis also confirmed that Vps34 knockdown increased LC3-II levels (Figure 5E). Notably, the effect was lost when cells were treated with the lysosomal inhibitors Baf or CQ (Figure 5E), indicating that Vps34-induced LC3-II accumulation is the result of impairment to autophagosome degradation, rather than enhanced autophagosome synthesis. These data, in agreement with those recently reported [55], confirm that LC3 lipidation is independent of Vps34 activity. Jaber et al [55] also reported that LC3 protein self-aggregation may partially account for LC3-positive vesicle accumulation in Vps34-null cells. Interestingly, a highly selective Vps34 inhibitor, PIK-III, also strongly induces accumulation of LC3-II/autophagosomes [56]. Although Vps34 is critical in PI(3)P generation, as we confirmed in Figure 5F, for autophagosome formation [7], it has been suggested to be dispensable for the process [57-59], but required for autophagosome maturation since Vps34 is critical in endosomal/lysosomal trafficking [53, 55] (see Discussion for more details). The knockdown efficiency of Vps34 and mTOR was also confirmed by qPCR (Supplementary Figure 2), in addition to immunoblot (Figure 5D). Our data, consistent with these previous findings, reveal that Vps34 inhibition by DKI results in defective autophagosome-lysosomal fusion, suggesting a mechanism by which DKI exerts an inhibitory effect on autophagy.

### DKI treatment induces cell death and inhibits cells proliferation

**Figure 6:**
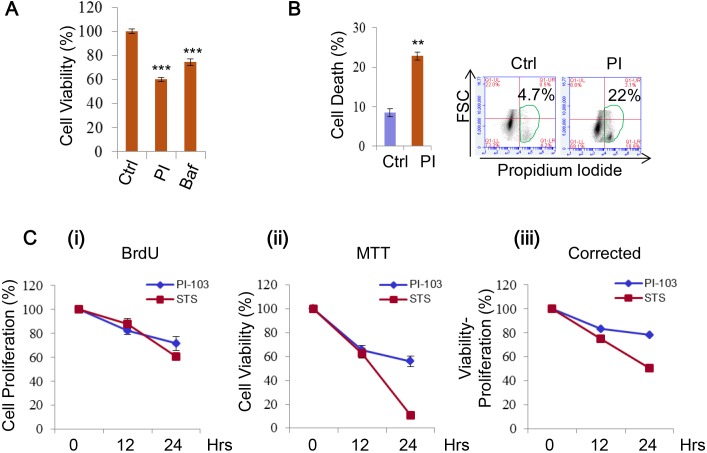
PI-103 treatment induces cell death and inhibits cell proliferation **A.** RT4 cells were treated with vehicle, PI (0.1 uM) or Bafilomycin A1 (Baf, 10 nM). Cell viability was measured via ATP assay kit (Promega) (*n* = 6). Data are shown as mean ±sd ***: *P* < 0.001. **B.** RT4 cells were treated with vehicle or PI (0.5uM) (24 hours) and cell death was measured using propidium iodide staining in flow cytometry (*n*= 3 per treatment). Data are shown as mean ±sd. **: *P* < 0.01. **C.** HeLa cells were treated with vehicle (as a negative control), PI (5uM) or staurosporine (STS) (1uM) (as a positive control) for the times indicated. Cell proliferation was measured by BrdU assay (i) and viability by MTT assay (ii). Viability readings were then corrected by subtracting results from the BrdU assay (iii) (*n* = 6 per condition). Data are shown as mean ±sd.

DKI treatments are known to be able to induce cell death, hence are attractive for tumour therapy [25, 26, 28, 31, 33]. We found the viability of RT4 cells to be sensitive to PI-103 treatment (Figure 6A, Supplementary Figure 3), thus used the cells for further cell death assays. The cells also showed a significant increase in cell death with the drug (Figure 6B). As the difference between the viability loss and cell death extent was notable, we reasoned this could be the result of additional factors that are encompassed by cell viability measurements, such as growth and proliferation, because both cell growth and proliferation are positively regulated by the PI-3K/mTOR pathway [15]. We confirmed this in HeLa cells treated with PI-103 by simultaneously comparing cell proliferation and viability. Over the time course, a decline in both measures was seen (Figure 6C (i-ii)), and the cell death shown by correcting viability for proliferation was still observable (Figure 6C (iii)). Thus, DKI treatments induce cell death, and also suppress cell proliferation.

### Cell death induced by DKI is independent of apoptosis

**Figure 7:**
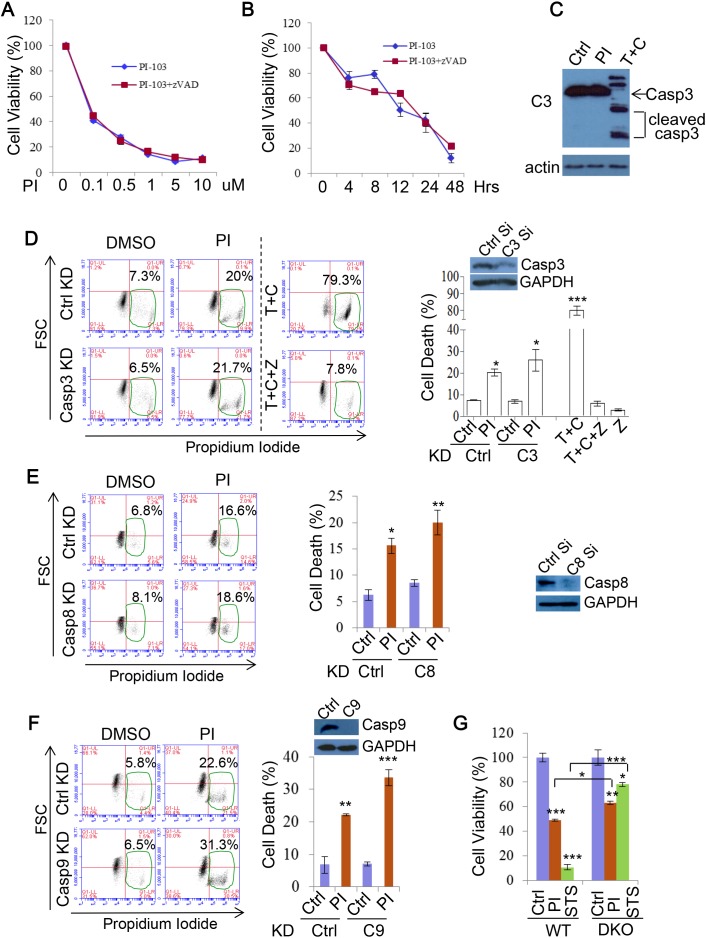
PI-103 induces cell death independent of apoptosis **A.** RT4 cells were treated with a series of concentrations of PI-103 (PI) alone, or with pan-caspase inhibitor zVAD-fmk (zVAD) (20uM) for 24 hours. Cell viability was measured via ATP assay kit (Promega) (*n* = 6 per condition). Data are shown as mean ±sd (note that the error bars are not visible due to minor variations). **B.** RT4 cells were treated with PI (0.5uM) with or without zVAD (20uM) for the time periods indicated, and cell viability was measured via MTT assay (*n* = 6 per condition). Data are shown as mean ±sd. **C.** HeLa cells were treated with vehicle, PI (5uM) or TNF (30ng/ml)+cycloheximide (CHX, 30uM) (as a positive control) for 20 hours and analysed by immunoblot with the indicated antibodies. **D.** HeLa cells were transfected with control or caspase-3 siRNA for 24 hours then treated with vehicle or PI (5uM) for 48 hours. Cell death was measured with propidium iodide staining (*n* = 6 per condition). TNF+CHX±zVAD was used as additional controls for apoptosis inhibition. Data are shown as mean ±sd. ***: *P* < 0.001, *: *P* < 0.05. Immunoblot was used to verify knockdown efficiency. **E.** HeLa cells were transfected with control or caspase-8 siRNA for 24 hours then treated with vehicle or PI (5uM) for 20 hours. Cell death was measured with propidium iodide staining (*n* = 6 per condition). Data are shown as mean ±sd. **: *P* < 0.01. Immunoblot was used to verify knockdown efficiency. **F.** HeLa cells were transfected with control or caspase-9 siRNA for 24 hours then treated with vehicle or PI (5uM) for 48 hours. Cell death was measured with propidium iodide staining (*n* = 6 per condition). Data are shown as mean ±sd. Immunoblot was used to verify knockdown efficiency. **G.** WT MEF or Bax/Bak DKO MEFs were treated with vehicle, PI (3uM), or STS (1uM) for 24 hours. Cell viability was measured via ATP assay kit (Promega) (*n* = 12 per condition). Data are shown as mean ±sd.

Previous studies, based on the fact that DKI induces production of apoptosis markers, suggested that DKI drug treatments execute cell death via the caspase-mediated apoptosis pathway [28, 60]. However, it was not clear if inhibition of apoptosis suppresses DKI-induced cell death. In order to further test this, we applied the pan-caspase inhibitor zVAD-fmk (zVAD) to PI-103 treatments. Unexpectedly, zVAD afforded no protection from PI-103 mediated cytotoxicity, suggesting that this effect was independent of apoptosis (Figure 7A). This phenomenon was not altered by extending the timeframe of the treatment to 48 hours (Figure 7B, Supplementary Figure 4). As apoptosis is mediated by caspase cleavage and activation, we explored caspase-3 cleavage in response to PI-103. Cleaved caspase-3 was not detectable in the cells treated with PI-103 for 20 hours, in contrast to the apoptosis inducing combination treatment of TNF and cycloheximide (CHX) (Figure 7C). Likewise, genetic knockdown of caspase-3 afforded no cell death protection to HeLa cells from PI-103 treatment, unlike that provided by the pan caspase inhibitor zVAD to TNF+CHX treatment (Figure 7D). To fortify this observation, we also assessed the effect of knocking down caspase-8 (Figure 7E) and caspase-9 (Figure 7F) on PI-103 induced cell death, and consistently saw no alleviation to the toxicity. Interestingly, it is noted that caspase-9 knockdown consistently caused more rather than less cell death in the cells treated with PI-103 (Figure 7F). Caspase-9 was suggested to regulate autophagy activity, and its inhibition blocks autophagy through modulating lysosomal pH and acidic-dependent cathepsin activity, thus augumenting cell death [61]. Finally, we investigated differences in viability between WT and apoptosis defective Bax/Bak DKO MEFs with PI-103 treatment, and compared these results with staurosporine (STS), an agent known to kill cells via apoptosis [62]. Whilst the loss in viability with STS was largely rescued in the DKO MEFs, the effect was much more marginal with PI-103, indicating a relatively minor role for apoptosis in the activity of this drug (Figure 7G). Together, these results suggest that apoptosis is not a major contributor to the viability loss and death associated with DKI treatments.

### DKI-induced cell death is independent of necroptosis

**Figure 8:**
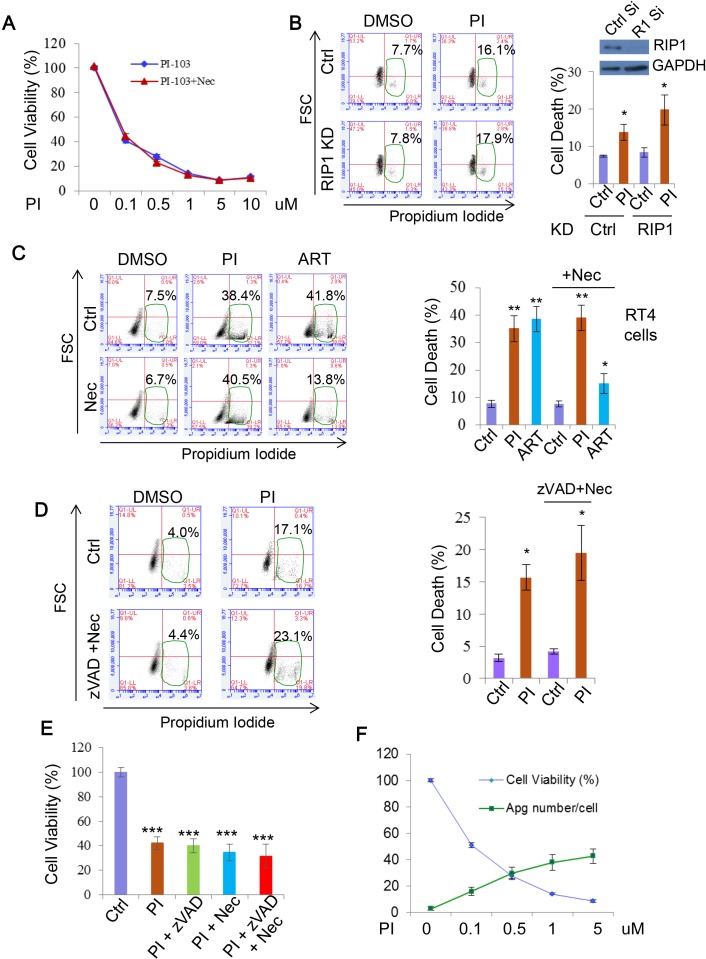
PI-103 induces cell death independent of necroptosis **A.** RT4 cells were treated with a series of concentrations of PI-103 (PI) alone, or with necroptosis inhibitor necrostatin-1 (Nec) (20uM) for 24 hours. Cell viability was measured via ATP assay kit (Promega) (*n* = 6 per condition). Data are shown as mean±sd (note that the error bars are not visible due to minor variations). **B.** HeLa cells were transfected with control and RIP1 siRNA for 24 hours then treated with vehicle or PI (5uM) for 20 hours. Cell death was measured with propidium iodide staining (*n* = 6 per condition). Data are shown as mean ±sd. *: *P* < 0.05. Immunoblot was used to verify knockdown efficiency. **C.** RT4 cells were treated with vehicle, PI (1uM), or artemisinin analogue, artesunate (ART) (25uM) with or without Nec (20uM) for 24 hours. Cell death was measured with propidium iodide staining (*n* = 6 per condition). ART (25uM) or/and Nec were used as additional controls for necroptosis inhibition. Data are shown as mean ±sd. **: *P* < 0.01. **D.** HeLa cells were treated with PI (5uM), zVAD (20uM) and Nec (20uM) for 24 hours. Cell death was measured with propidium iodide staining (*n* = 3 per condition). Data are shown as mean ±sd. **E.** RT4 cells were treated with vehicle, PI-103 (PI) (0.5uM), z-VAD (20uM) and nec (20uM) as indicated for 24 hours. Cell viability was measured with MTT assay (*n* = 6 per condition). Data are shown as mean ±sd. ***: *P* < 0.001. **F.** RT4 cells were treated with concentrations of PI as indicated (24 hours). Cell viability was assessed with ATP assay kit (Promega) (*n* = 6 per condition)). Autophagosome (Apg) numbers were assessed in matching RT4 cells with GFP-LC3 by automated Cellomics microcopy (*n* = 12 per condition). Data are shown as mean ±sd.

As apoptosis did not appear to be a major effector of PI-103 cytotoxicity, we next explored the involvement of another programmed cell death pathway, necroptosis. A key positive regulator of this process is RIP1, meaning that inhibition of this protein also prevents necroptosis onset [63, 64]. We first assessed the effect of the widely used necroptosis inhibitor Necrostatin-1 (Nec) [63, 65] on PI-103 cytotoxicity. As with apoptosis, loss of necroptosis provided no rescue of cell viability with PI-103 treatment (Figure 8A). Similarly, genetic knockdown of RIP1 did not afford protection from cell death in the cells treated with PI-103 (Figure 8B). Finally, we compared the effects of Nec on cell death with PI-103 or the artemisinin-based drug artesunate (ART), which we have previously demonstrated to induce necroptosis in RT4 cells [66]. Whilst ART toxicity was largely attenuated by the addition of Nec, no such effect occurred with PI-103 treatment (Figure 8C). Taken together, these results indicate that DKI induced cell death is independent of necroptosis.

As neither apoptosis nor necroptosis suppression significantly alleviated DKI-induced cell death, we reasoned that it was possible one pathway could compensate for the other. However, synergistic use of zVAD and Nec had no effect on cell death (Figure 8D) or viability loss (Figure 8E) with PI-103 treatment. Based on this, we ventured that alterations to autophagy from the drug treatment may have a role. Indeed, over a series of PI-103 concentrations the extent of cell death correlated with the numbers of autophagosomes in cells (Figure 8F). Autophagy is involved in a number of intracellular pro-survival roles, such as toxin clearance and resistance against various stresses. Therefore, while appreciating that it is unlikely that DKI-induced cell death is only attributed to a single pathway, we reason that autophagy dysfunction may contribute to the cell death.

## DISCUSSION

In this study, we show that two dual inhibitors of mTOR and PI-3K, PI-103 and NVP-BEZ-235, inhibit autophagy, suggesting that this effect is common across all such agents. Interestingly, our data also strongly suggest DKI-induced cell death is independent of apoptosis and necroptosis, and inhibition of autophagic flux by DKI may contribute to its-induced cell death.

DKI appears to inhibit autophagy by blocking autophagosome maturation. It is believed that inhibition of mTOR induces autophagosome formation, while inhibition of Vps34 blocks autophagosome-lysosome fusion, resulting in decreased autophagic flux, and accumulations of non-degraded autophagosomes. This finding differs from previous observations using PI-103, which suggest the drug increases autophagy activity [28]. While this earlier work focused largely on the levels of LC3 and p62, our approach encompasses additional factors such as alternative autophagy substrates, lysosome status and autolysosome fusion. Although we consistently found PI-103 to cause impaired p62 degradation across all the cell types tested in this study, it is interesting to note that Fan et al. [28] report a decrease in p62 with PI-103 treatment in glioma cell lines (although it is unknown whether PI-103 alters p62 mRNA levels in the settings). Therefore, it is possible that the effect of PI-103 and DKI on autophagy flux may be influenced in part by cell type. Further investigation may be warranted to elucidate the effect of DKI on cell types of interest.

Although Vps34 is critical for generating PI(3)P in the process of autophagosome formation [7], our data show increased levels of LC3-positive vesicles/LC3-II levels in Vps34 knockdown cells. These data are wholly in agreement with recent reports with Vps34 knockout [55] and a highly selective Vps34 inhibitor [56] on a number of grounds. First, LC3 lipidation is independent of Vps34 activity [55] (see also in Figure 5); second, LC3 vesicle accumulation may be partially caused by LC3 protein aggregation in Vps34-defective cells [55]; third, Vps34 complex II is required for endocytic sorting (including sorting of hydrolytic enzymes to the lysosome/vacuole and early steps in the endocytic pathway), endosome maturation and autophagosome maturation [50-53] (defects in autophagosome maturation cause autophagosome accumulation); finally, despite the importance of Vps34 in autophagosome formation, it is dispensable for the process. A number of studies have shown instances of non-canonical Vps34 independent autophagy in mammalian cells [57, 58]. Recent estimates have suggested Vps34 is accountable for ~65% of PI(3)P production [67], with the remainder attributed to other sources such as the less well characterized Class II PI(3)K [68]. In addition, Rubinsztein and colleagues have reported that PI(5)P can be used for autophagosome formation, serving a compensatory role in the absence of PI(3)P [59]. Indeed, Juhasz et al. [69] previously showed accumulation of autophagosomes in ESCRT/Vps34 double mutants, which indicates that loss of Vps34 does not completely prevent autophagosome formation, suggesting PI(3)P may not be absolutely essential for this process. On the other hand, the lack of autophagosome accumulation in Vps34 single mutants indicate that ESCRT complexes are partially functional in the absence of Vps34 [69]. Therefore, DKI treatment appears to perturb Vps34 activity and autophagosome maturation, thereby leading to suppression of autophagy flux, which contributes to cell death.

Previously DKI-induced cell death was suggested to be apoptosis-dependent [28, 60]. However, it was unclear if inhibition of apoptosis blocks DKI-induced cell death. We observed PI-103 caused cell death even under conditions of apoptosis and necroptosis inhibition. The discrepancy may stem from the fact that previous research concluded apoptosis largely from increases in apoptosis marker expression, or different experimental settings such as cell types. Our approach, utilizing both chemical and genetic repressors of these cell death mechanisms, provides a robust analysis on this front. It is worth noting that we do not exclude the association of apoptosis (or necroptosis) with DKI treatment, but these pathways do not appear to be major determinants for DKI-induced cell death in our conditions. Although our data at current are largely speculative on this front, we believe that autophagy inhibition by PI-103 contributes to its induced cell death. Investigating this possibility may prove interesting ground for future research.

The PI-3K/mTOR dual inhibitors hold a promise for treating cancers [24, 25, 28]. Both PI-103 and NVP-BEZ-235 have been trialed in various tumours, and additional inhibitors continue to be developed and investigated [30, 31]. Our finding, which clarifies that the dual inhibitors also inhibit autophagy, is important since autophagy is critical for cellular energy and nutrition homeostasis, as well as cell survival [70]. Elevated levels of autophagy in tumours are also associated with improved stress resistance and toxin removal, which can form a barrier to medical treatment [71]. To counter this response, a number of studies have coupled chemotherapy or radiotherapy with inhibition of autophagy flux, and shown enhanced toxicity to tumours [72, 73]. Interestingly, dual PI-3K/mTOR inhibitors have also proven to boost the efficacy of such therapies [26, 27]. Our results highlight a mechanism by which these synergistic effects are achieved. Given that the dual kinase inhibitors of PI-3K/mTOR have been widely implicated in clinical treatment trials, this study may be important for their future medical application.

## MATERIALS AND METHODS

### Antibodies and reagents

Rabbit polyclonal antibodies: anti-LC3 (1:10,000) (Novus Biologicals, NB100-2220); anti-phospho-70S6K (Thr389) (1:1,000) (Cell Signaling, 9205); anti-Akt (pan) (1:1,000) (Cell Signaling, 4691); anti-phospho-Akt (Ser473) (1:1,000) (Cell Signaling, 4060); anti-ULK1 (pan) (1:1,000) (Cell Signaling, 8054); anti-phospho-ULK1 (Ser757) (1:1,000) (Cell Signaling, 6888); anti-phospho-4E-BP1 (Thr37/46) (1:1,000) (Cell Signaling, 2855); anti-Atg12 (1:500) (Cell Signaling, 4180); anti-RIP1 (1:1,000) (Cell Signaling, 3493); anti-caspase 3 (1:1,000) (Cell Signaling, 9662); anti-Cleaved caspase 3 (1:1,000) (Cell Signaling, 9661); anti-caspase 8 (1:1,000) (Cell Signaling, 9746); anti-caspase 9 (1:1,000) (Cell Signaling, 9504); anti-LAMP1 (1:1,000) (Cell Signaling, 9091); anti-mTOR 7C10 (1:500) (Cell Signaling, 2983); anti-PI3 Kinase Class III (Vps34) (1:500) (Cell Signaling, 3358); anti-actin (1:2,000) (Sigma, A2066). Goat polyclonal antibody: anti-cathepsin D (1:200) (Santa Cruz, sc-6486). Anti-mouse monoclonal antibodies: anti-GAPDH (1:5,000) (Ambion, AM4300); anti-p62 (1:1,000) (BD, 610833); anti-tubulin (1:10,000) (Sigma, T9026). Bafilomycin A1 (Baf) (19-148) was purchased from Millipore. Chloroquine (CQ) (C6628), 3-methyladenine (3-MA) (M9281), rapamycin (Rap) (R0395), staurosporine (STS) (S5921), cycloheximide (C4859), artesunate (ART) (A3731) and necrostatin-1 (Nec) (N9037) were purchased from Sigma. zVAD-fmk (zVAD) (627610) was a product of Merck. PI-103 (528100) was from EMD4Biosciences, and NVP-BEZ-235 (10565) was purchased from Cayman Chemical. LY294002 (LY) (9901) was purchased from Cell Signaling Technology, and Human TNF-α was from Invitrogen (Sino Biological, 10602-HNAE-5). siRNAs were from Invitrogen (Ambion) or Dharmacon.

### Cell culture

Bax/Bak WT and double-knockout (DKO) MEFs, used in our previous study [41], RT4-D6P2T (RT4) schwannoma cells [66], purchased from Sigma (93011415), and HeLa cells were cultured in DMEM (D6046) media with 10% fetal bovine serum (FBS) (12133C) (Sigma).

### siRNA transfection

Cells were split to 60-80% confluence and incubated in antibiotic-free DMEM containing 10% FBS. siRNAs were transfected with Lipofectamine 2000 (Invitrogen) as per the manufacturer's instructions. siRNAs were used at a final concentration of 50nM. Non-targeting siRNA was the control siRNA. Cells were maintained in 10% FBS DMEM media without antibiotics for 24 hours post transfection, and then incubated in DMEM with 10% FBS for further 24-48 hours after medium change. Human RIP1 siRNA (#J-004445-07) and caspase-8 siRNA (#J-003466-16) were obtained from Dharmacon. Human siRNA sequences: FRAP1 (mTOR): (Sense: 5′-GGGCAUGAAUCGGGAUGAU-3′) (Invitrogen); PIK3C3 (Vps34): (Sense: 5′-GGGAAGAGAGAACAAAAGA-3′) (Invitrogen); caspase-3: (Sense: 5′-UGGAUUAUCCUGAGAUGGG-3′) (Invitrogen); caspase-9: (5′-Sense: GGUUCUCAGACCGGAAACA-3′) (Invitrogen).

### Cell viability assay

1. ATP assays. Cell survival was determined with Cell Titer-Glo Luminescent Cell Viability Assay kit (Promega, G7571) to measure ATP levels according to the manufacturer's instruction. Briefly, 100ul of Cell Titer-Glo reagent was added to the culture medium. Cells were placed on a shaker for 5 min and then incubated at room temperature for 10 min. The SPECTRA Max M5 reader was used for Luminescent reading.

2. MTT assays. MTT was purchased from Invitrogen. Briefly, 10ul of a 12mM MTT stock solution was added to culture medium and incubated at 37°C for 4 hours. Medium was exchanged with 100ul DMSO, mixed by pipetting, and placed on a plate shaker for 10 min. Absorbance was read at 562nm and a reference measurement at 650nm using the TECAN GENios V4.62-07/01 microplate reader (Tecan, Reading, UK) and XFLUOR4 Version V 4.51 software (Tecan).

### Cell proliferation assay

Cell proliferation was measured using BrdU Cell Proliferation Assay kit (Cell Signaling, 6813) and performed following the manufacturer's instructions. Absorbance was read at 450nm using the TECAN GENios V4.62-07/01 microplate reader (Tecan, Reading, UK) and XFLUOR4 Version V 4.51 software (Tecan).

### Flow-cytometry cell death assays

Cells were trypsinised and washed twice with cold PBS, then resuspended in 1x binding buffer (10mM HEPES, pH 7.4; 140mM NaCl; 2.5mM CaCl_2_) at 1×10^6^ cells/ml. We transferred 100ul of cells to a FACS tube, added 5ul propidium iodide (Sigma) and incubated for 15 min at room temperature. We added 200ul of 1xbinding buffer into each tube and analysed by flow cytometry.

### Immunocytochemistry

Cells were washed with PBS twice before fixation with 4% paraformaldehyde for 10 min. After a further three PBS washes, the fixed cells were permeabilised with 0.5% Triton in PBS for 10 min. Cells were blocked in blocking buffer (1% BSA, 1% heat inactivated goat serum in PBS) for 30 min at room temperature, and incubated with primary antibodies overnight at 4°C. Cells were subjected to three 10 min PBS washes, incubated with secondary antibodies for 30 min, then followed by an additional three 10 min PBS washes. Slides were mounted with DAPI (3ug/ml).

### mRFP-GFP-LC3 assay

HeLa cells stably expressing mRFP-GFP-LC3 were treated with compounds at the indicated concentrations. After 24 hours, cells were fixed in 2% PFA for 5 min. Cellomics (Arrayscan VTI) was used to score green and red vesicles. Green vesicles are considered to be autophagosomes and red vesicles are considered to be both autophagosomes and autolysosomes. The number of autolysosomes was achieved by subtracting the number of green vesicles from that of the red vesicles.

### Analysis of autophagosomes/vesicles

In experiments requiring a precise assessment of vesicle number, the number of vesicles per cell in GFP or RFP-positive cells was determined. Approximately 100 cells per sample were counted for triplicate samples, as described previously [41] or otherwise methods are described in each figure legend. All coverslips were scored with the observer blinded to the identity of the slides.

### Acridine orange staining

Following drug treatment incubation, cells were stained with 2.5μg/ml acridine orange (Fisher) for 15 min at 37°C and washed twice with PBS. Live cell imaging was performed on a Leica TCSSP8 with a 1.4nm 63x objective. The argon laser at 488nm was used for excitation with emission collected simultaneously at both 505-570nm and 615-754nm.

### LysoSensor green staining

Following drug treatment, cells were stained with 1uM LysoSensor Green DND-189 (Fisher) for 30 minutes at 37°C. The media was exchanged and then live cell imaging was performed by confocal microscopy using an excitation wavelength of 488nm.

### Subcellular fractionation

Cell fractionation was performed following Jahreiss et al [74]. Briefly, cells were harvested and pellets washed once in PBS, then resuspended in fractionation buffer (10mM HEPES pH7.9, 10mM KCl, 1.5mM MgCl_2_, 0.1mM EGTA, 0.5mM DTT, 0.5mM PMSF and 10ug/ml proteinase inhibitor). This mix was passed through a 21-gauge needle 10 times and kept on ice for 45 min. After removing some of the lysate (total fraction), samples were centrifuged (30 min, 16,000 × g, 4°C). The supernatant (cytosolic fraction) was collected. The pellet (membrane fraction) was washed twice with fractionation buffer (7 min, 16,000 × g, 4°C) before running with SDS-PAGE.

### qRT-PCR analysis

RNA isolation was performed with TRIzol reagent as instructed (Invitrogen). For qPCR analysis, 1ug RNA was reverse transcribed (Applied Biosystems, Paisley, UK) using cycles of 25°C (10 min), 37°C (120 min) and 85°C (5 min). cDNA templates were then used in qPCR with LightCycler 480 DNA SYBR Green I Master kit (Roche) in LightCycler 480 II system (Roche). All primers were from Sigma and used at 0.5uM. Actin was used as a control to normalize the data. Actin primers: 5′-ACTGGCATCGTGATGGACTC-3′ (forward) and 5′-TCAGGCAGCTCGTAGCTCTT-3′ (reverse); LC3-B primers: 5′-ACGATACAAGGGTGAGAAGCA-3′ (forward) and 5′-GTCCGTTCACCAACAGGAAG-3′ (reverse); p62 primers: 5′-AGATGAGGAAGATCGCCTTG-3′ (forward) and 5′-GGCATCTGTAGGGACTGGAG-3′ (reverse); mTOR primers: 5′-TAAGAAAACGGGGACCACAG-3′ (forward) and 5′-TGAGAGAAGTCCCGACCAGT-3′ (reverse); Vps34 primers: 5′-TCCTTGATGGTTGATGCAAA-3′ (forward) and 5′-CAGCAAAAAGAGCATGGACA-3′ (reverse).

### Quantification of autoradiographs

To quantify protein band density, the relevant specified bands were analysed using PhosphoImage software. The relative value was computed.

### Statistics

T-test was used and P-values were determined by unconditional logistical regression analysis by using the general loglinear option of SPSS 9.1 software (SPSS, Chicago, IL) (***, *P* < 0.001, **, *P* < 0.01, *, *P* < 0.05, NS, not significant).

## SUPPLEMENTARY MATERIAL FIGURES


