# Supplementary material for: Dual PI-3 kinase/mTOR inhibition impairs autophagy flux and induces cell death independent of apoptosis and necroptosis
Source: Oncotarget. 2016 Jan 22;7(5):5157–75. doi: 10.18632/oncotarget.6986 (PMC4868678; doi:10.18632/oncotarget.6986)
Supplement: Supplementary file 1 [file oncotarget-07-5157-s001.pdf]

Dual PI-3 kinase/mTOR inhibition impairs autophagy flux and induces cell death independent of apoptosis and necroptosis

Supplementary Material

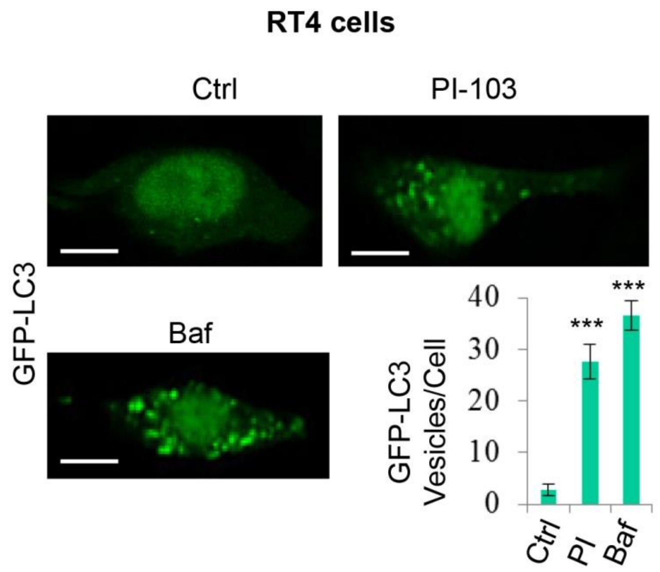

Supplementary Figure 1: PI-103 treatment induces autophagosome accumulations in RT4 cells

GFP-LC3 was transfected into RT4 cells. After 16 hours, cells were treated with vehicle and PI-103 (PI) (1uM) for 20 hours, and Baf (100nM) for 4 hours. The number of GFP-LC3 vesicles per cell were counted (n=30 cells per condition). Data are shown as mean ±sd. ***: $P<0.001$. Scale bar: 20um.

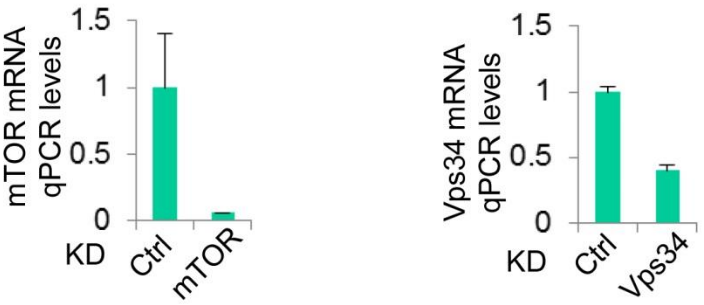

Supplementary Figure 2: Confirmation of mTOR and Vps34 knockdown

qPCR was used as another measure of knockdown efficiency of mTOR and Vps34 siRNA transfection (see Figure 5). Experiments were performed in triplicate, with n=3 per experiment.

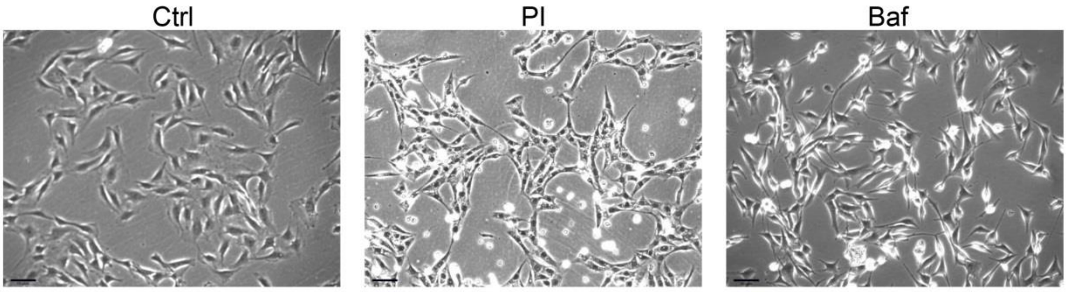

Supplementary Figure 3: Inhibition of autophagy flux reduces cell viability

RT4 cells were treated with vehicle, PI-103 (PI) (0.1 uM) or Bafilomycin A1 (Baf, 10 nM). Representative phase contrast images were acquired. Scale bar: 65um.

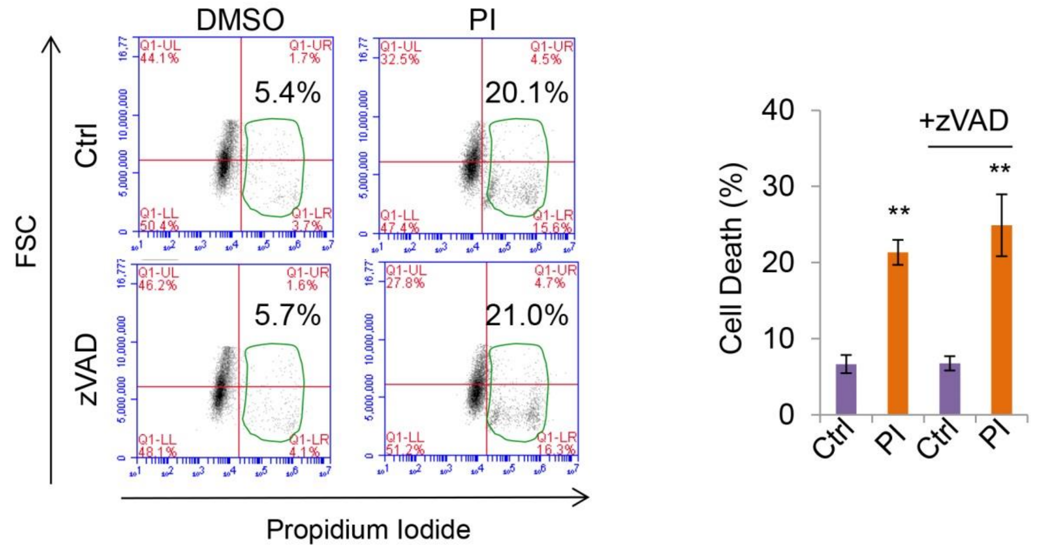

Supplementary Figure 4: PI-103 induces cell death independent of apoptosis

HeLa cells were treated with vehicle or PI-103 (PI) (5uM) ±zVAD (20uM) for 48 hours. Cell death was measured with propidium iodide staining in flow cytometry (n=6 per condition). Data are shown as mean ±sd. **: P<0.001.
